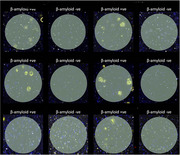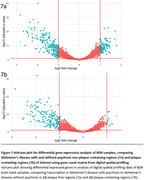# Applying Digital Spatial Profiling of the Transcriptome to Elucidate Disease Mechanisms of Psychosis in Alzheimer’s disease

**DOI:** 10.1002/alz.086517

**Published:** 2025-01-03

**Authors:** Jacob Thomas‐Hegarty, Katie Lunnon, Ehsan Pishva, Adam Smith, Luke Weymouth, Michael Eyres, Lucy Frost, Gayle Marshall, Eleanor Platt, Byron Creese

**Affiliations:** ^1^ University of Exeter, Exeter United Kingdom; ^2^ University of Exeter, Exeter, Devon United Kingdom; ^3^ Maastricht University, Maastricht Netherlands; ^4^ Medicine’s Discovery Catapult, Alderly Edge, Cheshire United Kingdom; ^5^ Medicines Discovery Catapult, Aldrerley Edge, Cheshire United Kingdom; ^6^ Medicine’s Discovery Catapult, Alderley Edge, Cheshire United Kingdom; ^7^ Brunel University London, London United Kingdom

## Abstract

**Background:**

Psychosis occurs in 30‐40% of individuals with AD. New insights into disease mechanisms may lead to novel pharmacological targets and treatments. Previous studies have focused on bulk tissue analysis with limited results. Digital spatial profiling (DSP) is a new technique for spatial analysis of RNA or proteins in fixed tissue. It allows quantitative profiling with spatial complexity to be collected from samples in a non‐destructive manner. In this pilot study we used DSP to compare whole transcriptome data in amyloid beta and non‐amyloid beta regions in participants with and without psychosis (AD+P; AD‐P).

**Method:**

Six post‐mortem brain samples from prefrontal cortex were provided by the Kings College London Brains for Dementia Research (BDR) brain bank. Frozen and formalin fixed, paraffin embedded (FFPE) sections were supplied in order to test the platform on each type. Psychosis positive and negative groups were selected based on Neuropsychiatric Inventory (NPI) assessments. Samples were hematoxylin and eosin (H&E) stained as well as stained with fluorescent antibodies for AT8, NeuN, SYTO13 and Aβ. Regions of interest (ROIs) are selected based on morphology markers and tissue morphology (see Figure 1 for Amyloid ROI selection).

**Result:**

H&E staining revealed the frozen samples to be too badly degraded so the analysis was conducted on FFPE sections. AT8 staining showed widespread tau pathology to the extent that it was not possible to confidently select non‐tau ROIs. Analysis of Aβ plaque containing and Aβ plaque free regions, comparing AD+P and AD‐P groups, found 314 differentially expressed genes in plaque free regions, and 172 differentially expressed genes in plaque containing regions (Figure 2). Of these 172 genes, 28 were not differentially expressed in plaque free regions, forming a plaque‐specific signature of genes differentially expressed in AD+P.

**Conclusion:**

This pilot study demonstrates the potential of the NanoString GeoMx™ DSP platform as an innovative spatial transcriptomics methodology for investigating AD+P with the potential to uncover differentially expressed genes that may be missed by bulk RNA sequencing studies. FFPE sections appear to be optimal. Analysing earlier stage disease and more sections per subject may help with better differentiation of tau and non‐tau ROIs.